# Comparison of plasma endothelin levels between osteoporotic, osteopenic and normal subjects

**DOI:** 10.1186/1471-2474-6-49

**Published:** 2005-09-20

**Authors:** Hasan Hilmi Muratli, Levent Çelebi, Onur Hapa, Ali Biçimoğlu

**Affiliations:** 1 3^rd ^Orthopaedics and Traumatology Clinic, Ankara Numune Education and Research Hospital, Talatpaşa Bulvarı, Sıhhiye, Ankara, Turkey

## Abstract

**Background:**

It has been demonstrated that endothelins (ET) have significant roles in bone remodeling, metabolism and physiopathology of several bone diseases. We aimed to investigate if there was any difference between the plasma ET levels of osteoporotic patients and normals.

**Methods:**

86 patients (70 women and 16 men) with a mean age of 62.6 (ranges: 51–90) years were included in this study. Patients were divided into groups of osteoporosis, osteopenia and normal regarding reported T scores of DEXA evaluation according to the suggestions of World Health Organization. According to these criteria 19, 43 and 24 were normal, osteopenic and osteoporotic respectively. Then total plasma level of ET was measured in all patients with monoclonal antibody based sandwich immunoassay (EIA) method. One-way analysis of variance test was used to compare endothelin values between normals, osteopenics and osteoporotics.

**Results:**

Endothelin total plasma level in patients was a mean of 98.36 ± 63.96, 100.92 ± 47.2 and 99.56 ± 56.6 pg/ml in osteoporotic, osteopenic and normal groups respectively. The difference between groups was not significant (p > 0.05).

**Conclusion:**

No significant differences in plasma ET levels among three groups of study participants could be detected in this study.

## Background

The endothelins (ET) are a family of 21-aminoacid peptides consisting of endothelin-1 (ET-1), the related peptides ET-2 and ET-3 [1]. In addition to being among the most potent vasoconstrictor agents known, ET have been found to possess a wide range of pharmacological activities on different tissues [1-3]. The close proximity of cells on the bone surface to vascular endothelial cells exposes bone cells to endothelial cell products such as the polypeptide ET. It is well recognized that ET play an important role in bone metabolism [4-8].

The receptors for ET on the osteoclasts, osteoblasts and their intracellular signal systems were predominantly found out by detailed in vitro and in vivo studies [3,8,9]. ET-A and ET-B receptor subtypes are expressed in bone cells. Stimulation of phospholipids turnover and activation of tyrosine kinases are used by ET as a major way while transducting the intracellular signals [3,9]. It was also demonstrated that osteoblasts, osteoclasts and osteocyts contain measurable amount of ET [10,11].

It was shown that ET regulates bone blood flow in the intact vascularized bone preparations [12]. However ETs' effects are not limited with their vasoactivity in the bone tissue. ET stimulates proliferation of the capillary endothelial cells [2,13-15], osteoblasts and osteoprogenitor cells [3,16]. They also stimulate differentiation of osteoprogenitor cells to osteoblasts [3,4,6].

Osteoblastic activity is increased by ET as this effect is demonstrated by stimulation of synthesis of collagen and non-collagen proteins [6] as well as osteocalcin and osteopontin messages in bone tissue [17].

It was demonstrated that ET has also certain interactions with 1,25 dihydroxyvitamin D3. Upon in vitro observations it has been addressed that ET together with vascular endothelial growth factor (VEGF) and 1,25 dihydroxyvitamin D3 may have an in vivo activity on bone formation and remodeling process [7].

There are some controversial reports regarding the effects of ET on bone resorption. They clearly inhibit motile process of the osteoclasts [5,8]. Osteoclastic bone resorption is inhibited by ET with similar doses that produce vasoconstriction [5]. Furthermore ET inhibits parathyroid hormone secretion in the parathyroid adenoma cells [18]. However along with these antiresorptive effects it was shown that they lead to prostaglandin (PG) [19,20] and interleukin-6 (IL-6) [21] related stimulation of resorption.

There are also controversial reports about ET effects on the mineralization process of bone. It is thought that mineralization is inhibited by ET through the stimulation of ET-A receptor [22,23]. On the contrary blockage of ET-A receptors are reported to cause osteopenia in experimental studies [24].

In the basis of above mentioned effects of ET in the bone, we thought that ET may have an important role in the physiopathology of osteoporosis and according to our investigation there is no epidemiologic study available in the literature, in particular to investigate the associations between ET and bone mineral density. We aimed to find out if there was any difference of the ET plasma levels between osteoporotic and normal people.

## Methods

### Groups of patients

242 patients who were over 50 years of age were referred by us to examine in our hospital's radiology department just for screening by dual energy X-ray absorbsiometry (DEXA) during from March to June 2004 were invited to the present study as soon as the results of DEXA were obtained. Patients with systemic diseases (diabetes, hypertension, renal disease, or clinical manifestation of atherosclerosis or known another diseases) and patients with abnormal laboratory results (regarding routine hemogram parameters and routine biochemical test) were planned to excluded. Other exclusion criteria were receival of any medication for osteoporosis previously or another drug in the last 3 months before the study and smoking or drinking alcoholic beverages for at least 48 hours before the blood sample receival. Presence of any anamnestic or clinical signs of osteoporosis (pain and previous fractures) and presence of any differences from reference values of the biochemical markers of bone remodeling in the patients with normal bone density according to our accepted criteria described below was accepted as another exclusion criteria.

The study was performed cross-sectionally. After first interview 12 of 242 invited patients refused to participate in the study. 44 patients who had known systemic diseases (diabetes, hypertension, renal disease, or clinical manifestation of atherosclerosis) and 22 patients who had received drugs in the last 3 months before the study were excluded at the beginning. 24 patients who received any treatment for osteoporosis before the study were also excluded. 16 participants who were evaluated as normal regarding bone mineral densitometry evaluation but who had any anamnestic or clinical signs of osteoporosis (pain and previous fractures) were also excluded from the study.

Remaining 124 patients who accepted to join our study were informed of the nature of the study. Consent was obtained from each participant. Then all patients were analyzed on clinical and biochemical basis. Then systemic blood pressures were measured in all other participants and 16 patients were excluded because of high blood measurement. Then Complete blood count and biochemical profiles including routine biochemical tests and biochemical markers of bone remodeling were assessed and 20 patients were excluded because of the pathological findings in this analysis. Although they were instructed not to use, 2 patients who smoked or drank alcoholic beverages in the period of 48 hours before the blood sample collection were excluded from the study.

At the end of these initial evaluation procedures remaining 86 patients were included in this study according to our accepted criteria. There were 16 males and 70 females. Mean age was 62.6 (ranges: 51–90) years. Patients were divided into 3 groups regarding reported T scores in DEXA evaluation. Consistent data base regarding young normal value and the population standard deviation were used in order to calculate T score. T-scores less than -2.5 on either total lumbar spine or total hip were accepted as osteoporosis, while scores between -1 and -2.5 were accepted as osteopenia and scores above -1 were accepted as normal according to the suggestions of World Health Organization (WHO) [25]. According to suggested criteria of WHO [25] 19 of 86 were normal, 43 were osteopenic and 24 were osteoporotic. All patients' demographic and anthropometrical characteristics were noted.

### Methods

After the insertion of a teflon cannula into the antecubital vein all subjects remained recumbent for at least 30 minutes. Then 5 ml of venous blood was withdrawn in vacutainer K_2_-EDTA plasma tubes from all patients 8 hours after overnight fasting. Blood samples were centrifuged immediately for 15 minute at 2000 × g. Then plasma samples were stored frozen at -80°C until EIA.

We had quantified the total amount of human ET fasting plasma level by using commercially available Endothelin-1 EIA (Endothelin EIA Kit, Catalog No:583151, Cayman Chemical Company, Michigan, USA) following instructions of the manufacturer. This immunometric assay is based on a double-antibody 'sandwich' technique and permits endothelin measurements within the range of 0–250 pg/ml, typically with a limit of detection of 1,5 pg/ml. Monoclonal anti ET-1 antibody in this kit had a cross reactivity of 100% with ET-2 and 100% with ET-3. Samples were assessed with no prior purification.

The intra- and inter-assay coefficients of variation of the method were 5 and 6%, respectively.

In this method monoclonal antibody specific to endothelin and acetylcholinesterase: Fab' Conjugate (AChE:Fab') bind to different epitopes on the Endothelin-1 molecule and forming sandwich. This sandwich is immobilized on the plate so the excess reagents are washed away. The concentration of analyte is detected by measuring the enzymatic activity of the AChE by adding Ellman's Reagent which contains the substrate for AChE. Addition of Ellman's Reagent produces a yellow-colored product which can be measured spectrophotometrically. The intensity of the color is directly proportional to the amount of bound conjugate which in turn is proportional to the concentration of the Endothelin.

Enzyme immunometric analysises were run twice from the same sample (in Düzen Laboratories Chain, Ankara, Turkey).

### Data analysis

Data analyses were done by SPSS for Windows version 11.5. Prior to the analysis, all the data were examined for accuracy of data entry and fit between their distributions and the assumptions of univariate analysis. To improve pairwaise linearity and to reduce the extreme skewness and kurtosis, the z score for all variables was computed. It was found that all dependent variables are normally distributed.

Data was analyzed using two-way analysis of variance (two-way ANOVA) to assess statistical significance. Independent variables were group of subject (osteoporotics, osteopenics and normals), and gender (male and female); dependent variable was endothelin value.

This analysis was also used to compare weight, height and body mass index parameters between the groups of subjects.

By using the formula y = y'-b.(x-x') (y: new endothelin, y': old endothelin, b: regression coefficient x: weight or height or body mass index or age, x': mean weight or height or body mass index or age) we had made endothelin independent of weight, height and body mass index because these variables were treated as covariate variables. Osteoporotics, osteopenics and normals were compared for statistical significance in terms of endothelin levels using one-way ANOVA test after controlling for covariates. Two-way ANOVA test was used to confirm the difference of endothelin level between the males and females for each group separately after controlling for covariates. A value of p < 0.05 was considered as significant.

Pearson Product Moment Correlation coefficient analysis was used to detect relation between age, body mass index, height, weight, T scores and endothelin levels. All values were given as mean ± S.D.

## Results

Age, gender and anthropometrical parameters of the groups were summarized in Table 1.

**Table 1:** Demographical and anthropometrical characteristics of the groups.

	**Number of cases**	**Gender**		**Age (years) Mean ± S.D.**	**Height (cm) mean ± S.D.**	**Weight (kg) mean ± S.D.**	**BMI (kg/m^2^) mean ± S.D.**
						
		**M**	**F**				
**Osteoporotics**	24	4	20	65.95 ± 9.4^a^	153.29 ± 7.69^b^	65.79 ± 12.4^c^	28.02 ± 5.14^a^
**Osteopenics**	43	7	36	62.60 ± 7.9	156.09 ± 6.48	75.90 ± 11.72	31.19 ± 4.68
**Normal**	19	5	14	58.20 ± 7.6	160.0 ± 13.27	83.15 ± 11.5	33.01 ± 6.61

^a^: There is a significant difference between normals, osteopenics and osteoporotics (p < 0.01)

^b^: There is a significant difference between normals, osteopenics and osteoporotics (p < 0.05)

^c^: There is a significant difference between normals, osteopenics and osteoporotics (p < 0.001)

Average systemic blood pressure of study participants was 126 ± 8 (Mean ± S.D.) mmHg and 75 ± 9 mmHg for systolic and diastolic levels.

### Endothelin fasting plasma levels

**Figure 1:**
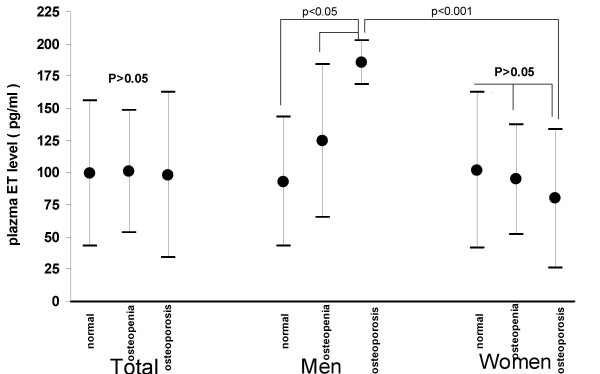
Plasma ET levels (mean ± S.D.) before the adjustments for age, weight and height of each group are presented diagrammatically.

Endothelin fasting plasma levels were found comparable in both runs of analysises. Unadjusted ET total plasma levels were a mean of 98.36 ± 63.96 pg/ml in osteoporotic group, a mean of 100.92 ± 47.2 pg/ml in osteopenic group and a mean of 99.56 ± 56.6 pg/ml in normal group. The difference between groups was not significant. (p > 0.05) (Figure 1)

### Levels according to gender

In men with osteoporosis mean unadjusted ET level was 185.7 ± 17.2 pg/ml and this was significantly higher than in osteopenic men (124.8 ± 59.6 pg/ml) and in normal men (93.0 ± 50.1 pg/ml) (p < 0.05). In women there was not any significant difference between groups (normal:102.0 ± 60.7 pg/ml, osteopenics: 94.7 ± 42.7 pg/ml, osteoporotics: 79.9 ± 53.8 pg/ml, p > 0.05). (Figure 1)

Independent of osteoporotic status mean ET level was significantly higher in men (130.1 ± 58.7 pg/ml) than women (91.5 ± 50.2 pg/ml). (p < 0.05)

In osteoporotics mean ET level was significantly higher in men than in women. (p < 0.001). In osteopenic men mean ET level was higher than women but it was not significant (p > 0.05). In normal group mean ET level was lower in men than in women but it was not significant too (p > 0.05).

### Levels according to age

When adjusted to age, ET levels did not differ significantly between groups. (p > 0.05)

### Levels according to anthropometrical parameters

Osteoporotics had significantly lower values of weight than normals and osteopenics. (p < 0.001) Osteoporotics were significantly shorter than normals and osteopenics. (p < 0.05) As a result of these osteoporotics had significantly lower body mass index than normals and osteopenics. (p < 0.01)

After separately adjustments to body weight, length and body mass index, ET levels did not differ significantly between groups. (p > 0.05)

Regardless of groups there was no significant correlation between neither weight nor height and ET values. (p > 0.05)

In normal groups no correlation was found between weight, length, body mass index and ET levels. (p > 0.05) In osteopenic patients we have found negative correlation of weight (r:-0.36, p < 0.05), body mass index (r:-0.45, p < 0.01) and ET values. In osteoporotics we have found positive correlation of height and ET levels. (r:0.41, p < 0.05)

### Other correlation studies

Regardless of groups and in osteopenics and osteoporotics there was no correlation between vertebral or hip T scores and ET values (p > 0.05) but a correlation between hip T scores and ET values of normal group (r:-0.5, p < 0.05).

## Discussion

After demonstration of ETs' important effects on bone tissue it was begun to be considered that they may have certain roles in the physiopathology of some clinical entities [26,27]. As a matter of fact Tarquini et al. measured the circulating ET-1 levels of patients with Paget Disease in which the bone turn over is extremely increased and they found out that ET-1 level was significantly increased in patients with Paget Disease when compared to controls [27]. So they believed that ET-1 may have a role in the physiopathology of this disease and it could be used as a marker.

We investigated if ET had a role in the physiopathology of osteoporosis. We found out that comparison regardless of gender among osteoporotics, osteopenics and normals and comparison of female osteoporotics, osteopenics and normals yielded no significant differences regarding plasma ET levels. In addition regardless of groups (according to suggested criteria of WHO [25]) no correlation was found between vertebral or hip T scores and ET values. Although plasma ET levels of osteoporotic men were found significantly higher than normal men we believed that it could be speculative to make conclusion with these findings because there were only 4 osteoporotic men in the series.

Studies about the men osteoporosis demonstrated us that although total estradiol levels do not change substantially over life in men, bioavailable estradiol levels decrease to 50% of the levels in young men in the older ages. It is thought that this decline in bioavailable estradiol levels may be the major cause of bone loss in elderly osteoporotic men [28,29]. In previous laboratory studies it was shown that estrogens down regulated ET-1 both through the secretion from the vascular endothelial cells and m-RNA expression levels [30,31]. Upon these observations we believed that possible reason for higher plasma ET levels of osteoporotic men then the normal men in our study may be because of the lower bioavaliable estrogen concentration of the males in these ages and as a result possible decrease of estrogens effect in down regulation in ET and consequently increase of ET amount and effects. In fact bone loss is more accelerated in women after menopause as a result of a decline in circulating estrogens levels then the men in the same ages period [32]. However considering presence of no difference in the plasma ET levels between the osteoporotics, osteopenics and normals in the women population of our study participants it is not possible to say same mechanism is true for women regarding the ET and estrogens interaction.

Regarding osteoporosis physiopathology there are many effects of ET which can cause bone resorption and inhibition on the mineralization process as follows. ET-1's effect through the PG system [19,20] and IL-6 expression [21] are in favor of stimulating bone resorption. Recently it was described that expression of mRNA of PG endoperoxide G/H synthase is stimulated by ET-1 and this stimulation is a way of leading increase of PGE-2 production [20]. Tatrai and Stern [19] showed that ET-1 modulates the intracellular calcium signalization of PGE-1 and if the cells confronts with ET-1 then PGE-1 comes out. As a result ET-1 causes bone resorption depended with PGs.

Hierl et al. [21] showed that ET-1 has a dose dependent stimulatory effect on IL-6 expression in human osteblastic cell (HOC) cultures. IL-6 leads to bone resorption potently and it's this action was described with detailed in vivo and in vitro studies. And ET-1s' bone resorptive effect in the cell culture environment was mainly attributed to its effect on the IL-6 expression stimulation.

There are also reports about inhibitory activity of ET on the mineralization process of bone. Hiruma et al. [22] demonstrated that calcium deposition into the bone cells is decreased by ETs in rat calvarial osteoblast like cell cultures. They concluded that mineralization process in the osteoblasts may be inhibited by ET-1 through the ET-A receptor. Inoue et al. [23] also reported similar findings.

Considering findings of reports cited above which are in favor of ET's stimulatory effect on bone resorption and inhibitory effects on the mineralization process on bone tissue we thought ET can be the important peptide in the osteoporosis physiopathology and we thought we can find differences in the plasma ET levels between osteoporotics and normal subjects. But our findings do not support this idea.

According to manufacturer's instructions normal levels of ET-1 in human plasma are below the detection limit of the kit which we used; therefore purification and concentration of the sample is necessary for accurate measurement of ET-1 levels. Considering 100% cross reactions of this kit for all ET subtypes including ET-1, ET-2 and ET-3 and performing our analysis without prior purification process it should be addressed that our measurements reflects total endothelin measurements, not ET-1 alone. We did not perform purification because manufacturer states that samples can be assayed with no prior purification in general and they suggest performing purification process only for samples containing low concentration of endothelin (0–50 pg/ml). They also state that samples must be >50 pg/ml in order to be assayed accurately with this kit and all average ET levels of our groups was already in this range. In addition all samples obtained from both normals and pathologics regarding bone densitometric evaluation were evaluated with the same method, without prior purification, and all obtained measurements were within the detection range of this kit (0–250 pg/ml). So we believe that in the evaluation of our results and comparison of these findings with other studies these points should be taken into consideration.

Our study has certain limitations. Firstly a number of study participants were relatively small because osteoporosis is a complex disease, known to be affected by many factors such as age, gender, adiposity, menopause etc. We believe that subsequent studies should be performed with larger number of participants especially for men. In addition although we only included patients with no known disease and with normal laboratory findings in the routine evaluation tests and normal blood pressure, it was not possible to know with this limited evaluations if these patients had any disease which are not possible to diagnose with our screening tests for this study and which may also cause changes of plasma ET level as mentioned and referenced below.

Although it is known by many studies that [27,33,34] increased level of ET concentrations can be detected in the plasma as a result of overproduction of ET released from pathologic tissues and/or due to hypervascularization associated with the lesion in osseous or non-osseous pathologies, it can be thought that systemic circulation may thus not entirely reflect local changes in the bones. So in the substantial studies ET concentrations at the site of osteoporotic bone tissue should also be evaluated by biopsies.

## Conclusion

According to our findings there is no significant difference between the ostoeporotics, osteopenics and normals regarding plasma ET levels. We think that particularly estrogens, prostaglandins and interleukin levels should also be measured in similar study designs in order to discuss the role of ET in osteoporosis and the difference of ET levels between males and females.

## Competing interests

The author(s) declare that they have no competing interests.

## Authors' contributions

HHM conceived of the study, and participated in its design and coordination and helped to draft and write the manuscript.

LÇ carried out the bone mineral density and immunoassay measurement organization.

OH carried out the immunoassay study organization and statistical analysis.

AB participated in the design of the study and helped to draft the manuscript.

All authors read and approved the final manuscript.

## Pre-publication history

The pre-publication history for this paper can be accessed here:


